# Integrating Big Data, Artificial Intelligence, and motion analysis for emerging precision medicine applications in Parkinson’s Disease

**DOI:** 10.1186/s40537-024-01023-3

**Published:** 2024-10-30

**Authors:** Laura Dipietro, Uri Eden, Seth Elkin-Frankston, Mirret M. El-Hagrassy, Deniz Doruk Camsari, Ciro Ramos-Estebanez, Felipe Fregni, Timothy Wagner

**Affiliations:** 1 Highland Instruments, Cambridge, MA USA; 2 Boston University, Boston, MA USA; 3 U.S. Army DEVCOM Soldier Center, Natick, MA USA; 4 Center for Applied Brain and Cognitive Sciences, Tufts University, Medford, MA USA; 5 Department of Neurology, UMass Chan Medical School, UMass Memorial, Worcester, MA USA; 6 Mindpath College Health, Isla Vista, Goleta, CA USA; 7 Mayo Clinic, Rochester, MN USA; 8 University of Illinois Chicago, Chicago, IL USA; 9 Spaulding Rehabilitation/Neuromodulation Lab, Harvard Medical School, Cambridge, MA USA; 10 Harvard-MIT Division of Health Sciences and Technology, Cambridge, MA USA

**Keywords:** Big Data, Parkinson’s disease, Artificial Intelligence, Wearables, Prediction, UPDRS, Clustering, Precision medicine, Noninvasive brain stimulation

## Abstract

One of the key challenges in Big Data for clinical research and healthcare is how to integrate new sources of data, whose relation to disease processes are often not well understood, with multiple classical clinical measurements that have been used by clinicians for years to describe disease processes and interpret therapeutic outcomes. Without such integration, even the most promising data from emerging technologies may have limited, if any, clinical utility. This paper presents an approach to address this challenge, illustrated through an example in Parkinson’s Disease (PD) management. We show how data from various sensing sources can be integrated with traditional clinical measurements used in PD; furthermore, we show how leveraging Big Data frameworks, augmented by Artificial Intelligence (AI) algorithms, can distinctively enrich the data resources available to clinicians. We showcase the potential of this approach in a cohort of 50 PD patients who underwent both evaluations with an Integrated Motion Analysis Suite (IMAS) composed of a battery of multimodal, portable, and wearable sensors and traditional Unified Parkinson's Disease Rating Scale (UPDRS)-III evaluations. Through techniques including Principal Component Analysis (PCA), elastic net regression, and clustering analysis we demonstrate how this combined approach can be used to improve clinical motor assessments and to develop personalized treatments. The scalability of our approach enables systematic data generation and analysis on increasingly larger datasets, confirming the integration potential of IMAS, whose use in PD assessments is validated herein, within Big Data paradigms. Compared to existing approaches, our solution offers a more comprehensive, multi-dimensional view of patient data, enabling deeper clinical insights and greater potential for personalized treatment strategies. Additionally, we show how IMAS can be integrated into established clinical practices, facilitating its adoption in routine care and complementing emerging methods, for instance, non-invasive brain stimulation. Future work will aim to augment our data repositories with additional clinical data, such as imaging and biospecimen data, to further broaden and enhance these foundational methodologies, leveraging the full potential of Big Data and AI.

## Introduction

A new era of Big Data is dawning on clinical research and clinical care. New sources of data including portable and wearable clinical sensors, mobile healthcare technologies, and smartphones offer the potential to augment scarce and costly clinical measurements with continuously recorded signals reflecting a wider scope of patient activity. One of the key challenges is how to effectively integrate these new sources of data, whose relation to disease processes are often not well understood, with multiple classical clinical measurements, and the expertise that clinicians have developed based on decades-long classical medical tradition-based definitions of disease, progression, treatment, and understanding of outcomes. While this challenge is present across different medical specialties, it is exemplified in emerging approaches in PD clinical research and treatment development, where engineers are increasingly proposing innovative methods for continuous patient monitoring in various settings, but innovations struggle to gain traction in clinical practice, where clinicians still prefer traditional measures such as questionnaires and semi-quantitative clinical scales even though the latter offer a fraction of the resolution and advantages of new technologies. This paper presents an approach to addressing this challenge, illustrated through an example in PD management.

The paper is organized as follows. In the remainder of this section, we introduce PD and traditional clinical management approaches, highlighting their limitations and the need for novel strategies. We also explore the potential of emerging data sources, such as sensor data from wearable devices processed with AI and Big Data techniques, to enhance clinical practice, and we address the barriers to their adoption. In Sect. “Problem Definition”, we highlight the key challenge, and this paper’s primary focus, of integrating these innovations with established clinical methods, crucial for fully harnessing their potential in advancing PD management. In Sect. “Existing Solutions”, we review the current state-of-the-art and show that existing approaches remain fragmented and do not fully address this core issue. Section “Proposed Solution” introduces our solution: IMAS, which integrates multiple sensing technologies with conventional PD clinical assessments. Compared to existing approaches, IMAS provides a more comprehensive and multi-dimensional view of patient data, offering deeper clinical insights and enhanced potential for personalized treatment strategies. Additionally, IMAS supports advanced decision-making through its use of machine learning and Big Data, allowing for integration into routine clinical care. Its design balances innovation with established practices, positioning IMAS as a significant step forward. Sect. "Elaboration" presents experimental results from a study of 50 PD patients, demonstrating IMAS’s effectiveness in addressing the integration challenge. Finally, Sect. "Conclusion" offers concluding remarks and recommendations for future research.

### PD significance and traditional clinical approaches to disease management

PD is the second most common neurodegenerative disorder globally [1] affecting ~ 8.5 million people worldwide [2] and poses a significant public health challenge. PD prevalence is increasing worldwide [2]. Disability due to PD is increasing faster than for any other neurological disorder [3]. In 2019, PD resulted in 5.8 million disability-adjusted life years, an increase of 81% since 2000, and caused 329,000 deaths, an increase of over 100% since 2000 [4]. The economic burden of PD is also increasing (in the United States (U.S.), the estimated growth is from $51.9B in 2017 to $79B by 2037 [5]).

PD is a long-term degenerative disease that impacts patients through decades and typically presents with a slow progression and mounting disability through time. It is characterized by progressive motor and non-motor symptoms that primarily stem from the degeneration of dopaminergic cells of the substantia nigra pars compacta. Motor symptoms include tremor, rigidity, bradykinesia, and postural instability, which are often accompanied by distinct neuroimaging findings and genetic polymorphisms [6]. The phenotypic expression of these symptoms exhibits substantial inter-individual variability, with each patient presenting a unique constellation of symptoms and rate of progression. The trajectory of symptom evolution is dynamic with a progressive variability that requires therapeutic adjustments. In addition, new motor and non-motor symptoms may arise during the progression of disease.

**Fig. 1:**
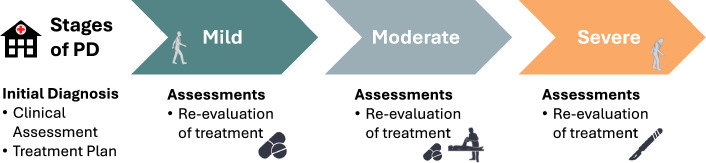
**PD continuum of care.** After an initial diagnosis, primarily based on a history and motor examination, and potentially supplemented by neuroimaging and L-Dopa challenge results, patients enter the care continuum. Symptoms are monitored periodically, and treatments are adjusted depending on patient response. Treatment depends on symptom type and severity and might include pharmacological, Physical Therapy (PT), neuromodulation, and/or surgical interventions. Continued assessments are a fundamental component of the PD care continuum

The continuum of care for PD patients (see Fig. 1) relies on a multi-disciplinary team that follows a series of critical steps that include initial assessments and diagnosis, treatment planning, treatment execution, and ongoing monitoring, which comprises subsequent assessments, follow-up evaluations, and ancillary support services. Most of these steps still heavily rely on clinical observation and trial-and-error approaches. This reliance introduces an additional dimension of variability (inter-observer) which stems from differences in the interpretation and evaluation of clinical findings among healthcare practitioners with varying degrees of expertise and experience.

Specifically, diagnosis and evaluation of disease severity and progression are still primarily based on subjective neurological exam findings of PD-characteristic motor symptoms. This approach entails several limitations, such as low sensitivity and high subjectivity, especially when exams are performed by professionals with a focus of practice beyond movement disorders such as primary care physicians or advanced practice providers. It is estimated that non-movement disorder specialists can make diagnosis errors upwards of ~ 30% of the time (e.g., diagnoses found incorrect upon autopsy examination or confirmed via longitudinal examinations) [7, 8], with similar limitations in continuing assessments [8–24]. These limited results may be explained by non-specialists’ limited exposure to PD and arguably due to their lack of ability to assess PD symptoms holistically and recognize disease patterns [10, 20]. While PD diagnoses and ongoing assessments by movement disorder specialists are more accurate [7, 23], data shows that a significant percentage of patients receive their diagnoses and/or continuing assessments from non-specialists [11, 12, 24–30]. Conversely, PD patients followed by neurologists have less risk of being placed in a nursing facility and less likelihood of death, yet less than 60% of PD patients receive any neurologist care [29, 31].

In clinical settings, PD severity and progression are assessed with subjective scales like the UPDRS, both classic and Movement Disorder Society (MDS) versions, with section III specifically evaluating motor symptoms. For example, the classic UPDRS-III assesses 14 clinician-rated motor endpoints (e.g., tremor at rest, gait, posture), scoring each on a scale from 0 to 4 based on severity of impairment (with a 0 being normal performance or symptom absence). Although widely used, UPDRS evaluations are lengthy and suffer from limited resolution and high intra- and inter-rater variability [23, 32–34]. Ultimately these limitations impact patient care [11, 12, 19, 26, 29] and limit therapy customization [34–36], while also imposing significant resource requirements on PD clinical trials (e.g., large sample sizes, high costs, false positives or negatives) [37].

PD has no known cure. Available treatments aim to manage symptoms and include pharmacological (e.g., L-Dopa), PT, neuromodulation (e.g., Deep Brain Stimulation (DBS)), and/or surgical (e.g., pallidal-thalamotomy) methods. Similar to assessments, the selection of treatment(s) mostly relies on clinical observation, and it is complicated by the heterogeneity in symptomology. Treatment adjustments for PD, including in medication dosages, DBS settings, and PT plans, often rely on trial and error to manage the disease's variable response. Personalized, tailored therapies are recommended to address PD’s complex, evolving symptoms, aiming for an individualized approach rather than a one-size-fits-all strategy [38]. However, in practice, personalized treatments often boil down to periodic adjustments based on clinical observations, comorbidities, and patient feedback.

While PD incidence and impact are growing, PD clinical care is also facing another problem, namely decrease in availability of trained neurologists. The aging global population, combined with the scarcity of neurologists in the U.S. and internationally, especially outside metropolitan areas [11, 12, 19, 22, 39–47], is likely to exacerbate the issues of inaccurate assessments and sub-optimal treatment plans, increasingly compromising clinical care. Furthermore, other specialists, such as physical therapists and occupational therapists with PD-specific training are even less available [31]. To address the above concerns, leading health organizations such as the MDS PD task force [10], the American Academy of Neurology [13], and other international bodies have issued recommendations for the development of new, objective assessment tools [12, 19]. Specifically, the MDS PD task force states that PD assessments should account for all patient data and be systematized “so that they can be reproducible between clinicians (essential in research studies) and applied by clinicians with less expertise” [10]. Additionally, these advancements could provide the basis for more customized therapeutic strategies for patients.

### Harnessing sensing technologies, Big Data, and AI for enhancing PD management

Addressing these challenges requires the development of new methodologies that not only overcome the limitations of traditional methodologies employed in the clinic, but also adapt to understaffed environments. In this context, developing new technologies is a strategy that can comprehensively improve the clinical care pathway. Portable and wearable sensors, mobile healthcare technologies, AI, and, more recently, Big Data are progressively being explored and used in PD and broader healthcare contexts [6, 48], offering a unique opportunity to address these multifaceted issues. Big Data techniques augmented by machine learning algorithms are particularly suited for addressing PD’s ‘high-dimensional’ complexity and therapeutic response. They can exploit data variability and heterogeneity to uncover patterns, correlations, and insights that are not apparent through traditional analysis approaches, increase accuracy of prediction models, and facilitate the segmentation or clustering of data (which might serve as foundation to develop new treatments). However, despite their potential, in PD, Big Data and AI approaches remain largely underexplored or confined mostly to foundational research rather than progressing to studies with a clear path to clinical applicability. Many solutions fail to specifically identify or target nodes within the clinical care continuum for integration, which hampers their clinical applicability. While specialist-based care will remain the gold-standard, integration of Big Data, AI, and sensing technology into clinical research and patient care holds enormous potential to augment this care. Additionally, these technologies can enhance the assessment, diagnostic accuracy, and efficiency of non-specialists (e.g., general practitioners or nurse practitioners), thereby improving patient outcomes and expanding access to effective treatment.

## Problem definition

As traditional clinical methods face limitations in managing complex diseases like PD, there is a rise in the use of wearable and portable devices as well as AI, contributing to the growing complexity and volume of data. This trend amplifies the challenge of integrating these new data sources with established clinical measures. Effectively merging these new technologies with established clinical practice is crucial to leveraging the extensive knowledge clinicians have accumulated through decades of research and patient management. Without such integration, even the most promising data from emerging technologies may have limited, if any, clinical utility. This paper addresses the core challenge of integrating data from these emerging technologies with traditional clinical data, with a focus on PD.

## Existing solutions

In this section, we review published studies that have utilized wearable/portable sensors, machine learning, and Big Data in applications for PD. Subsection “Sensor-based solutions” focuses on solutions based on camera-based, inertial, and force sensors, namely the sensing sources used by IMAS (but different than our approach, not used in an integrated manner). Subsections “Integration with machine learning algorithms for prediction of clinical scales” and “Integration with Big Data” focus on studies on machine learning and Big Data for predicting UPDRS-III scores or for clustering. Subsection “Summary” summarizes the state-of-the-art of existing solutions, highlighting their limitations.

### Sensor-based solutions

Several researchers have explored the use of sensor-based measurements to generate more accurate, less variable motor exams and to assess clinical findings with higher resolutions than classic clinical scales [49]. Wearable inertial sensors, such as accelerometers and gyroscopes, have been used to assess movement in PD patients (see [50, 51] for a review) and specifically to measure a body segment’s linear acceleration [34, 52–54] and angular velocity respectively, to indirectly characterize postural sway, gait, and tremor [53, 55–71]. For example, Cancela et al. used a network of accelerometers attached to patients’ limbs and belt to assess PD gait [58]; Lopane et al. assessed feasibility of using a waist-worn inertial sensor for discriminating between Levodopa-induced dyskinesias and physiological sway in PD patients [56]; and Hssayeni et al. used inertial sensors to measure PD patients’ tremor recorded during free body movements [72]. Cameras have also been used to assess PD. For example, Kahn et al. used a computer-vision approach to track and quantify index-finger motion during finger tapping [73]; Rocha et al. evaluated a Red Green Blue-Depth (RGB-D) camera (Microsoft Kinect) and found that among several quantitative gait parameters, the variance of the center shoulder velocity presented the highest discriminative power to distinguish between non-PD, On, and Off states [74]. Force plates have been used to assess PD [54, 75] via force and/or pressure measurements (e.g., body’s center-of-pressure (CoP)) to indirectly characterize balance and postural sway [76–79]. Examples of commercial, sensor-based systems include Kinesia (Great Lakes Neurotechnologies, Cleveland, OH) which integrates Electromyography (EMG) with data from accelerometers and gyroscopes, Physilog (Gait Up, Renens, Switzerland) which is an ambulatory system for body motion analysis though inertial sensors, Personal KinetiGraph (Global Kinetics) which is a wrist-worn device that provides a continuous measure of movement, Mobility Lab (Ambulatory Parkinson’s Disease Monitoring), and the Portable Motus System (Motus Bioengineering)—see also [80] for a review.

Most existing systems, including commercial systems, have primarily relied on a single sensing modality and/or focused on single disease signs and/or single body segments/joints [50–52, 80–84]. This approach has several issues. First, it does not capture the systemic PD disease state; instead, it only provides limited snapshots of single specific disease findings [34, 52, 80]. Second, each sensing modality suffers from specific technical limitations. For example, despite many advantages such as portability, low power consumption, and no need for a clear line of sight for measurements (as required by cameras), signals taken from accelerometers cannot uniquely characterize the spatial position of joints/body segments, which severely limits their exclusive use for comprehensive motor assessments [85]. In fact, accelerometers can only measure acceleration (along axes that are constantly changing when worn by a moving patient [85]), but velocity and displacement can only be estimated via integration, a process severely limited by drifts and non-zero fluctuating offsets [85]. Gyroscopes have similar issues [85]. Force plates can only characterize balance and postural sway indirectly [76–79] but cannot provide the detailed information on joint position or coordination [86, 87] necessary to fully assess posture and balance control, which in fact rely on multi-joint (e.g., ankle and hip [88]) coordination strategies [89, 90]. This severely limits assessments, as significant postural changes often occur in PD [91]. Furthermore, CoP-based metrics alone do not seem to correlate with UPDRS [55]. Cameras can directly record spatial positions from multiple body segments, from which velocity, acceleration, and higher order derivatives can be computed [92, 93]. However, limitations including line of sight requirements [94, 95], low positioning repeatability [94, 96, 97], noise due to background movement [98], sensitivity to reflections and/or lighting conditions, and the need for at least 2 cameras (and/or markers [99, 100]) to track the position of a body segment in 3 dimensions [92, 101] severely limit cameras’ use for biomechanical assessments [92–96, 102, 103]. Finally, metrics used by these systems are often designed ad-hoc and their correlation with standard clinical scales is unclear. This limits their clinical usability, arguably preventing the widespread adoption of sensor-based systems for PD assessment into clinical settings.

### Integration with machine learning algorithms for prediction of clinical scales

Several studies have explored the use of sensor-based measurements to predict PD patients’ UPDRS or MDS-UDPRS or their subscores. For example, Liu et al. predicted tremor subscores of MDS-UPDRS-III from automatically analyzed videos and reported an accuracy greater than 85% (N = 130) [104]. Metha et al. predicted bradykinesia, and postural instability and gait UPDRS-III subscores from videos of sit-to-stand tasks automatically analyzed (N = 32) via deep learning-based methods and reported that their models outperformed two clinician video-raters benchmarked against in-clinic assessments [105]. Parisi et al. used 3 wearable inertial sensors (mounted on chest, and thighs) to record patient movements during leg agility, sit-to-stand, and gait tasks for automatic assignment of the corresponding MDS-UPDRS-III subscores (N = 34) [106]. Safarpour et al. used 3 inertial sensors to collect data during two standing balance tasks (in the lab) and gait and turning (in daily life) and predict rigidity and postural instability and gait difficulties (PIGD) MDS-UPDRS-III subscores (N = 31) and reported that predictions were significantly correlated with the subscores (r = 0.49 and r = 0.61, respectively) [107]. As part of PERFORM European project, Cancela et al. used accelerometers (limbs, trunk, and belt) to record unconstructed, daily living activities to predict UPDRS-III bradykinesia scores (N = 20) and reported accuracy in the range of 70%-86% [58]. Pan et al. used smartphone 3D accelerometers to predict hand resting tremor and gait difficulty UPDRS-III subscores (N = 40; r = 0.74 and r = 0.79, respectively [108]). Exley et al. used force plate data to predict subscores of postural stability (r = 0.599; p = 0.014; R^2^ = 0.35) among other subscores (N = 42 patients and N = 43 controls) [109]. Islam et al. analyzed finger tapping tasks recorded from a webcam and predicted finger tapping MDS-UPDRS-III subscores (scale 0–4, N = 250), and reported a lower mean absolute error (MAE) of 0.58 points vs the MAE of 0.83 obtained as average across raters with various levels of expertise (but higher error compared to the most expert neurologists (MAE of 0.53)) [110].

Only a few studies investigated prediction of the total UPDRS-III (or MDS-UPDRS-III) score. Zia Ur Rehman et al. measured gait during a 2-min continuous walk over a 25 m oval circuit over multiple sessions (longitudinal assessments were made every 18 months up to 72 months) with an accelerometer positioned on the lower back to predict total MDS-UPDRS-III (for training (N = 70) and testing (N = 46) the average MDS-UPDRS-III was 37.56 (12.13) and 38.11 (13.38), respectively). Results showed that scores predicted with a convolutional neural network (CNN) model correlated (r = 0.82) with true values (MAE of 6.29 points) when making predictions from the 72 month data based on models developed from the 36 month data and subsequently tuned on the patients' 54 month data [111]. Eguchi et al. investigated whether videos of gait (with participants walking toward the camera, turning around, and walking away) analyzed with CNNs could predict total UPDRS-III score (and axial symptoms, bradykinesia, rigidity, and tremor subscores) (N = 74) [112]. They made predictions on 10% of the patients, based on models developed from 80% of the patients, and tuned on 10% of the patients. For the total UPDRS-III score, they reported varying MAEs for different score ranges: 0–10, 11–20, 21–30, 31–40, and 41–108. These MAEs ranged from 4.2 to 14.0, depending on the UPDRS-III score grouping, but with an overall R^2^ of 0.59. Lobo et al. predicted MDS-UPDRS-III from 59 features of gait extracted from a 10 m walk monitored with two accelerometers (wrist and lower back) and reported a Leave-One-Out Cross Validation (LOOCV) of 11.5 MAE (N = 74, average MDS-UPDRS-III score 40.92 (14.31) [113]. Sotirakis et al. measured walking (2 min) and postural sway (30 s, eyes-closed) using six inertial sensors and predicted MDS-UPDRS-III (N = 74, 7 visits spaced 3 months apart, average MDS-UPDRS-III 24.4 (12.0) at visit 1). Their best model obtained a root mean square error (RMSE) of 10.02 (0.88) with 29 features via fivefold cross validation [114]. Hssayeni et al. used two inertial sensors (wrist and ankle) to record free body/Activity of Daily Living (ADL) movements and using ensemble deep learning models predicted UPDRS-III and reported a statistically significant correlation between the clinical scores and the predicted scores (r = 0.79) and an MAE of 5.95 points via LOOCV on an ensemble of 3 models [115].

### Integration with Big Data

In PD, Big Data approaches remain relatively underexplored [6]. Among the few studies that have explored the use of wearable/portable sensors for recording motor behavior, a significant portion has focused on home assessments via consumer electronics for longitudinal evaluations. For instance, the i-Prognosis project employs smartphones and an app for longitudinal assessments aimed at aiding diagnosis and developing strategies for improving patients' quality of life [116]; Cohen et al. explored the use of a Pebble watch to measure metrics such as gait, activity level, nighttime activity, and tremor [117]; and Prince et al. showed how finger tapping and memory test data from the mPower database collected with smartphones could be used to monitor the longitudinal behavior of both PD patients and healthy subjects [118]. A few studies have investigated identification of PD subtypes via AI-based clustering techniques, but they have mostly focused on datasets different than motor symptoms, such as genetic and neuroimaging data, or on motor symptoms assessed via clinical scales (e.g., see [119–121]). As above, the few studies that have employed sensor-based data for clustering purposes have mainly aimed at developing methods for longitudinal evaluations via home-based or mobile technologies to quantify impairment and discriminate between pathological and healthy behavior. For example, Williamson et al. used supervised learning on wrist-worn accelerometer data in the U.K. Biobank (409 subjects including 218 PD subjects) to detect abnormalities in longitudinal assessments for symptoms early detection and severity tracking [122]. Surangsrirat et al. analyzed finger tapping data from the mPower database (N = 8,003 subjects) and using a K-means algorithm identified 3 clusters, each with different characteristics possibly related to PD severity (as measured by mPower survey data and PDQ-8 scores) [123]. Nguyen et al. used unsupervised algorithms to identify clusters from features extracted from inertial sensors data collected during gait and assessed their power to discriminate severity of impairment, as measured by the UPDRS-III “gait” and “postural stability” subitems (N = 119 PD subjects) [124].

### Summary

Prior art on sensor-based solutions aimed at improving PD care has mainly focused on specific types of portable/wearable sensors (e.g., camera, inertial, or force sensors) used in isolation, primarily for data capture and feature extraction to facilitate monitoring in research settings. Machine learning applications that predict UPDRS-III or MDS-UPDRS-III from this type of data have several limitations. Only a few studies predict the total UPDRS-III or MDS-UPDRS-III score, with the majority targeting specific dimensions of motor impairments, e.g., gait, thus omitting a full assessment of patient motor status [115]. Additionally, these applications often rely on feature engineering [111] and many are unclear on model tuning and/or generalization. Among Big Data analytics, the integration of portable/wearable sensors data primarily focuses on smartphones for home-based and longitudinal monitoring. Yet, the methodology for integrating these solutions into PD care remains unclear, posing significant challenges for data synchronization and patient care coordination. In summary, our analysis of the state-of-the-art indicates that while numerous studies offer valuable insights, they remain fragmented and fail to comprehensively address the fundamental challenge outlined in Sect. "Problem Definition", namely integrating emerging technologies with traditional clinical methodologies.

## Proposed solution

To address the challenge outlined in Sect. "Problem Definition"  and the fragmentation in current solutions described in Sect. "Existing Solutions", we propose the IMAS. Developed by our group, IMAS integrates sensor data collected via an array of multi-modal portable and wearable sensors with traditional clinical information using machine learning algorithms. IMAS processes locally collected sensor data, with potential for incorporating external datasets, to enhance the algorithms’ performances and broaden research and clinical capabilities (e.g., for data-guided treatment personalization). Specifically, IMAS was designed to: improve objectivity of clinical assessments by using quantitative, sensor-based measures which can be systematically collected during patient visits; predict traditional clinical assessments such as UPDRS-III; and aid treatment customization. Below, we detail the components and operation of IMAS, and discuss how it differs from existing methodologies through both technological innovation and conceptual advancement.

**Fig. 2:**
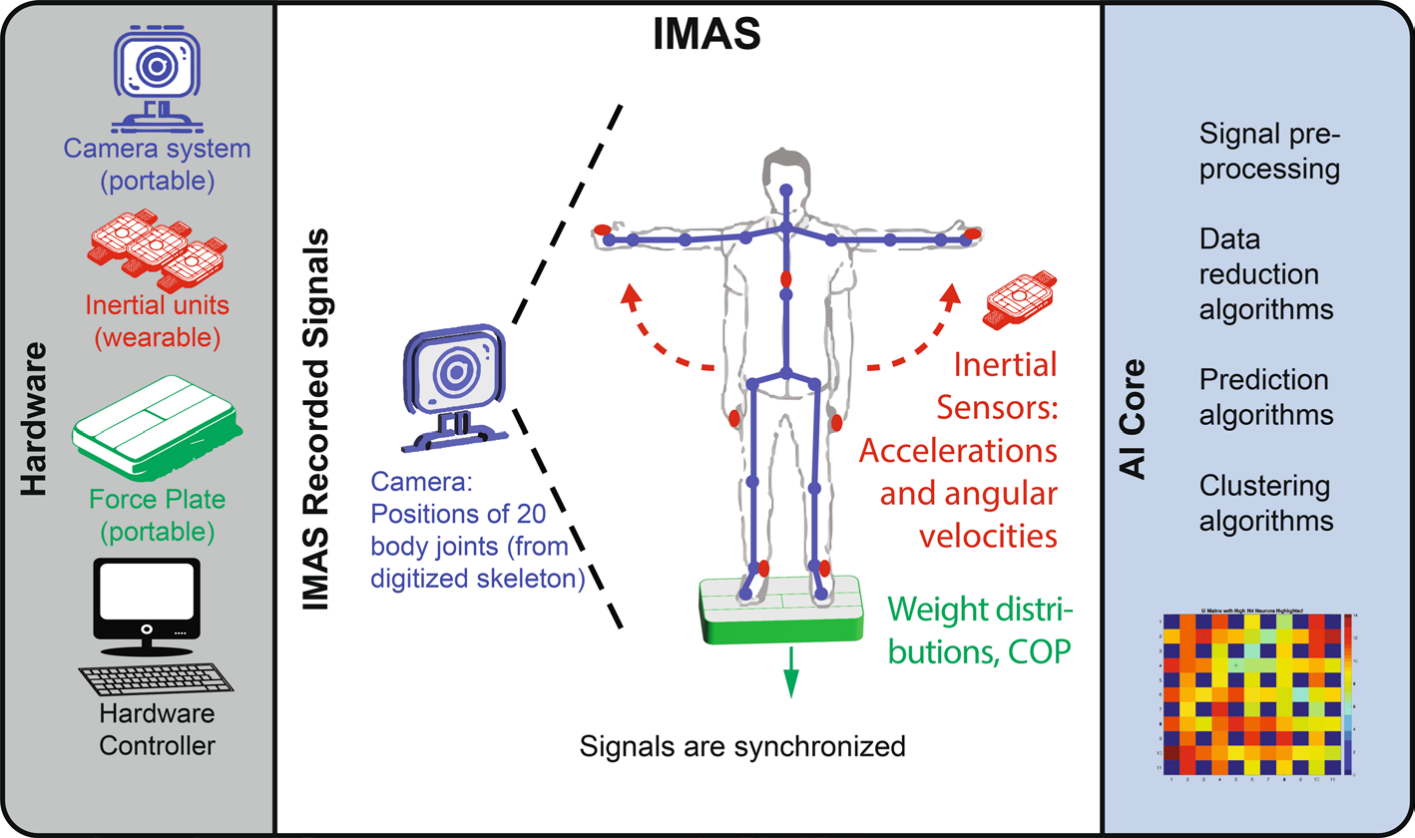
**IMAS.** During IMAS assessments patients are monitored with a battery of sensors, including camera-based, inertial, and force sensors. **IMAS recorded signals**: Integrating different sensor modalities allows recording the patient’s motor status and overcomes the limitations associated with using a single type of sensor. Notably, the camera-based system is equipped with a computer-vision software that generates a skeleton core of the patient and monitors the position of 20 or more joints in real-time. All signals are synchronized. **IMAS AI core**: The AI core is equipped with a battery of algorithms for off-line processing, including data reduction and machine learning. Parts of this figure are adapted from Fig. 5 in our paper [6] and Creative Commons licensed images

**Fig. 3:**
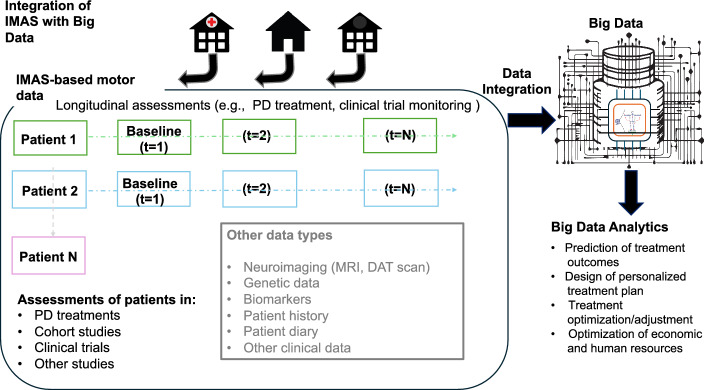
**Integration of IMAS with Big Data.** IMAS motor assessments can be performed in diverse settings (e.g., clinics, PT offices, or the patient’s home). We are building a database of IMAS data collected from PD patients at different times (“t” in the figure) and undergoing different treatments, including neuromodulation, PT or a combination thereof, as well as data from other patient cohorts with limited mobility (see Fig. 8). Engineered to facilitate systematic, quantitative data recording, along with software for automated data analysis, IMAS ensures the production of homogenous datasets. This homogeneity facilitates implementation of clinical protocols, enhances the comparability of results across clinical sites, and enhances statistical analysis for instance by reducing bias

IMAS includes multiple sensing modalities (3D motion capture camera, inertial sensors, and a force plate); a set of computational algorithms for data reduction, modeling, and prediction; and a patient-tracking database (see Fig. 2). It is postulated that movement disorder specialists assess PD motor systems in their entirety, with pattern recognition capabilities that non-expert clinicians lack [10, 20]. IMAS attempts to mirror this process by acquiring a broad picture of the disease state by combining multiple sensing modalities across multiple joints. However, it is not sufficient to simply collect data from multiple sensor types; a full picture of the disease state requires careful analysis to integrate and distill information across sensors. To this end, the sensing sources are coupled with algorithms for signal pre-processing and machine learning for data reduction and prediction/classification for a variety of purposes including clustering patients and predicting outcomes. While the IMAS was originally designed as an ancillary technology for objective motor evaluations to be coupled with a novel neuromodulation technology our group is developing for treating PD [125, 126], its algorithms can evaluate, track, and/or predict outcome of various treatments (e.g., PT administered as a single treatment or in combination with neuromodulation). Finally, the patient-tracking database, characterized by a Big Data architecture, allows recording and visualization of patient improvements through time as well as exploration of data clusters and identification of trends (e.g., groups of patients who respond well to a certain treatment) (see Fig. 3).

Five key design choices should be highlighted that incorporate both innovative and established approaches, distinctly positioning IMAS within the existing literature and enhancing its capabilities beyond those of existing systems within the literature: (1) combination of multi-modal sensors to overcome the limitations of each sensing modality and acquire a more comprehensive picture of the disease state across multiple joints; (2) prediction of clinical scales widely used in clinical practice [127] (UPDRS-III herein) to adhere to data representations that are readily interpretable by clinicians; (3) use of sensor-based metrics alongside predictions of traditional clinical scales data to allow further analysis such as classification/prediction; (4) a Big Data architecture that not only helps clinicians understand which treatments are likely to be most effective for patients with similar profiles but also supports personalized treatment planning and facilitates integration with current clinical practices and patient flow, ranging from Electronic Health Records (EHRs) to data fusion (including imaging data and biospecimens); and (5) a streamlined, modular, and robust design to enable IMAS to integrate new technologies, as well as facilitate use outside of clinical settings (e.g., the home).

Below, we present experiments conducted with IMAS, aimed at assessing: (1) the dimensional complexity of the IMAS dataset, specifically its variable diversity and structural intricacies; (2) the efficacy of predictive UPDRS-III modeling, utilizing elastic net regression for its robust variable selection capabilities, which leverage the intrinsic properties of the dataset; and (3) the dataset’s capability for segmentation into distinct clusters, as demonstrated through the use of a Self-Organizing Map (SOM), followed by hierarchical clustering. While these experiments are not exhaustive representations of IMAS full capabilities, they illustrate how a multi-modal sensor system, when integrated with a computational framework, can implement Big Data strategies to improve prognosis, treatment optimization, and care of PD, while also demonstrating how IMAS can be integrated into the PD clinical care pathway.

## Elaboration

### Methods

#### Experiments

Our dataset was collected as part of the baseline assessments for a randomized controlled trial investigating non-invasive brain stimulation for the treatment of PD (ClinicalTrials.gov Identifier: NCT01615718). Not all the patients examined herein entered the main trial, and the dataset was developed from the first 50 patients that underwent baseline assessments. Experiments took place at Spaulding Rehabilitation Hospital (SRH), Charlestown, MA. All procedures were approved by the Institutional Review Board of SRH, and written informed consent was obtained from all participants prior to participation.

As part of the study, subjects’ UPDRS-III motor scores (UPDRS Q18-31) were assessed and summed for the total score [128]. Evaluations were performed during ‘On’ periods (defined per [129, 130]). Subjects were then asked to perform a series of motor tasks designed to assess bradykinesia, ability to perform complex movements, tremor, postural instability, and gait, while their movements were tracked using our IMAS. For the foregoing analysis, IMAS assessments focused on subjects' most affected side.

Patients were asked to perform a series of 7 motor tasks: (1) elbow flexion–extension, (2) hand opening-closing, (3) sequence of hand opening-and-closing and elbow flexion/extension, (4) hand touch nose, (5) hand held still, (6) modified Romberg, (7) 10 m walks. The specific details of these tasks were completed as follows: 1A) continuous elbow flexion/extension movements: subject was instructed to move as fast as possible, keeping the wrist stable, palm up, beginning at level of waist/hip, going up to shoulder without touching it or overextending, and keeping the elbow stable but not pressed to the side [10 repetitions]; 1B) discrete elbow flexion/extension movements: similar to 1A), but stopping for 2 s at the end of each movement without letting the hand flop, and going as fast as possible in between; 2A) hand opening/closing at shoulder level: subject was instructed to fully open and close their hand fully in a fist (not clenching hard) as fast as possible, keeping the hand at the shoulder level (10 repetitions) starting with the hand open; 2B) hand opening/closing at hips/waist level: similar to the test described in 2A); 3) complex motor sequence involving multi-joint movements: subject was instructed to perform the hand opening/closing movements at the waist/hip and shoulder, and the flexion/extension movements as fast as possible in between with the hands open (10 repetitions); 4) hand-to-nose: keeping the arm/elbow at shoulder level, subject was asked to bring their hand (horizontal, palm down) almost to their nose without touching it and to extend it all the way to the side again, beginning with the arm outstretched and moving at their natural pace (10 repetitions); 5A) hand resting on table: subject was asked to rest their hand and forearm on a table, with the arm relaxed, for 30 s while visually fixating on the evaluator’s index finger swinging back and forth; 5B) hand resting in front of face: with arm/elbow at shoulder level, subject was asked to take their hand close to their nose and keep the hand there for 15 s while visually fixating on the evaluator’s index finger swinging back and forth; 6) balance test: with feet positioned in the middle of each side of a Wii board (i.e., feet about shoulder width apart) subject was asked to maintain an upright position for 15 s; test was performed twice, once with eyes open (while visually fixating on a pre-defined landmark) and once with eyes closed; 7) walking test: subject was asked to walk for 10 m at their usual pace (4 repetitions). Tests 1–5 were performed in seated position. Subjects wore hospital-provided, non-slip gripper socks throughout tests 6–7. Subjects’ motor performances during all tests were monitored by IMAS.

A subset of the 50 patients, N = 11, who were part of the main study’s placebo group had data available from a second session. In the second session, both IMAS and UPDRS-III data were again collected, about 1 week after the first session [131].

#### Sensor data collection

The IMAS version used in these experiments (see Fig. 2) included a commercial Kinect portable camera-based system (Microsoft, Redmond WA; 30 Hz sampling rate) [132–134], wearable three-axis gyroscope/three-axis accelerometer inertial measurement units (IMU) (64 Hz sampling rate), a portable force plate (Nintendo, Redmond, WA; 98 Hz sampling rate), and a remote controller for event marking (not shown in Fig. 2). The camera system included an embedded infrared sensor for measuring depth [135], i.e., recording in 3D, and commercial software for segmenting the human body from background, modeling the body as a 20-joint skeleton (hip center, spine, shoulder center, head, left and right shoulders, elbows, wrists, hands, hips, knees, feet, and ankle joints), and tracking 3D positions of the 20 joints [97, 136] (indicated as blue circles in Fig. 2).

The IMUs were attached to the subject’s body with Velcro straps or elastic cloth material that cuffed the body segment, with anatomical landmarks guiding the positioning. For example, in the IMAS assessments discussed herein, the primary IMU’s placement was as follows: for tests 1–5, an IMU was placed on the top side of the patient’s index finger; for the balance tests, it was positioned on the subject’s back, at the level of L5, near the body’s center of mass; for the walking tests, patient’s movement was tracked with two IMUs, one on L5 and another on the right ankle using the lateral malleolus as a landmark for the first 2 repetitions; for the last 2 repetitions, each ankle (right and left lateral malleoli) was tracked with a separate IMU. A remote controller allowed the experimenter to mark recordings; the marker signal was set whenever an event occurred (e.g., beginning or end of each motor task) and was null otherwise. The number of IMUs and locations for each test were chosen as a trade-off between time maximizing completeness of information, minimizing overall IMAS-testing duration, and minimizing number changes of sensor configuration (note, these assessments were part of a clinical trial that included other evaluations). All motor tests were tracked with the camera system, except for the walking tests. For the balance test, patients were asked to stand on the force plate. Throughout the experiments, camera, force plate, and subject’s chair (when used) were kept in fixed positions to minimize set-up times between sessions, prevent errors due to equipment re-positioning, and maintain consistency between participants. Custom C# routines were written to synchronize the recordings from all the IMAS sensors and the remote controller.

### Data analysis

#### IMAS metrics

IMAS metrics were extracted from the IMAS signals recorded during the above motor tests. For each test, the total task time was determined as the time from the first and last time the marker signal became positive. Then, the following metrics were calculated. For the elbow flexion/extension and hand-to-nose tests, wrist movements speed profiles *v* were calculated from the first order derivative of the 3D wrist trajectories smoothed with a 10 Hz low-pass FIR filter, segmented, and used to compute movement mean speed, max speed, duration, smoothness (ratio between mean speed and max speed), and number of movements, similar to [137, 138]. The path length traveled by the wrist in space was also calculated. For the hand opening/closing tests, angular velocity signals from the gyroscope (X_rot_, Y_rot_, Z_rot_) were filtered with a 4th order low-pass Butterworth filter (5 Hz cut-off). Metrics included movement time (total time divided by the number of movements) and inter-peak interval (interval between consecutive times when the hand was fully open, as marked by positive peaks in the angular velocity component X_rot_). Analysis of the complex movement focused on total time to complete the task. Resting and postural tremor were extracted from the accelerometer data recorded during the hand resting tasks. Resting tremor was calculated as the ratio of power in the 3–6 Hz band and total power, where power was evaluated with multi-taper spectral analysis from acceleration amplitude, which was calculated from the 3 components of acceleration [139–143] (other methods were also explored, i.e., mean of the power in the 3–6 Hz frequency band and mean of total power and calculation of both metrics using Fast Fourier Transform; a similar method was used to assess postural tremor (5-8 Hz)[143]). As for the balance tasks, the length of the path traveled by the subjects’ body CoP as measured by the board was calculated similar to [144]. Postural sway was further characterized with standard deviation of CoP components and axes length and area of an ellipse fitting CoP oscillations, calculated similar to [138]; also, the mean and peak values of jerk (first-order derivative of acceleration) amplitude were calculated from the acceleration measured by the IMU placed on L5 along the antero-posterior and medio-lateral directions similar to [55, 78] in order to characterize postural sway smoothness [55]. Separate values for the eyes open and eyes closed tests were calculated. For gait, besides total task duration, the following metrics were calculated from the IMU recordings after signals were filtered (4th order Butterworth low-pass filter, 5 Hz cutoff). For walks 1–2, movement smoothness was calculated as normalized jerk (mean jerk magnitude divided by mean speed [145]) where jerk amplitude was calculated from the first order derivatives of the filtered components of the signals recorded from the accelerometer mounted on L5, smoothed with a 4th order low-pass (5 Hz cutoff) Butterworth filter. For walks 3–4, the peaks of the Z_rot_ gyroscope signals (the angular velocity component where movements were most evident) were identified to assess when strides occurred; then, we calculated the distance between successive peaks (stride duration) and stride count. For all tests that required multiple movements, mean and standard deviation were calculated. Custom MATLAB routines were written to extract the metrics from the IMAS recordings.

#### Data reduction, UPDRS-III prediction, and clustering

PCA [146] was used to examine the correlation structure in the UPDRS-III and IMAS metrics and to estimate the effective dimensionality of both data sets. Each measure in each data set was standardized by removing its mean and dividing by its standard deviation and PCAs analyses were conducted for the set of UPDRS-III and IMAS measures, separately and together.

Elastic net regularization [147, 148] was used to identify a sparse set of predictors from the IMAS dataset and build linear regression models to predict the UPDRS-III. The elastic net penalty parameters were systematically varied using a grid search approach to find the best combination with highest R-squared value with the model Degrees of Freedom (DFs) capped at 50% of the patient group size. The number of DFs was capped to further reduce model complexity. Model performance was assessed with LOOCV to evaluate the model's predictive accuracy, quantified by R^2^ and MAE metrics. To further evaluate the methods generalization ability, we tested a model trained on day 1 observations to predict day 2 UPDRS-III scores from the IMAS metrics extracted from the dataset of N = 11 subjects (see above). Prediction errors were compared with published values of inter-rater and intra-rater variability of UPDRS-III [23].

Several clustering techniques were applied to the IMAS dataset to gain insights into its data structure. First, we employed t-Distributed Stochastic Neighbor Embedding (t-SNE). Then, clustering was performed using a SOM, followed by hierarchical clustering on neuron weights extracted from the SOM using the Ward method and a random forest classifier was used to assess the importance of different features with respect to the higher-level clustering. Experiments were conducted with different SOM grid sizes, number of epochs, and initial neighborhood sizes. For the hierarchical clustering with the Ward method, experiments were conducted with different numbers of desired groups. A dominance algorithm was used to evaluate feature dominance across clusters. Initially, one-way ANOVA identified significant mean differences among clusters for each feature (p < 0.05). Bonferroni-corrected post-hoc tests determined which specific pairs of clusters differed. For each feature within these pairs, the median value for each cluster was calculated, and a feature was deemed dominant in a cluster if it had the highest median value within that pair. Note, dominance was quantified by aggregating the counts of instances where a feature's median was the highest across its significant pairwise comparisons. Finally, the resulting clusters were input into the elastic net regression model to evaluate if they could enhance prediction accuracy. Then, these clusters were compared to those obtained using previously reported clinical subgroup calculations based on UPDRS-III score groupings of tremor-dominant, akinetic-rigid, and mixed subtypes as in [149]. Analyses were performed using custom routines written in MATLAB (Mathworks, Natick, MA).

### Results

Data from all 50 patients was analyzed (36 males, 14 females, mean age 64.5 yrs. (9.8), mean UPDRS-III 22.7 points (10.1) assessed during ‘On’ periods. IMAS evaluations were also conducted during ‘On’ periods [129]). For each subject and evaluation day, a total of 62 metrics descriptive of motor behavior was extracted from the IMAS recordings.

**Fig. 4:**
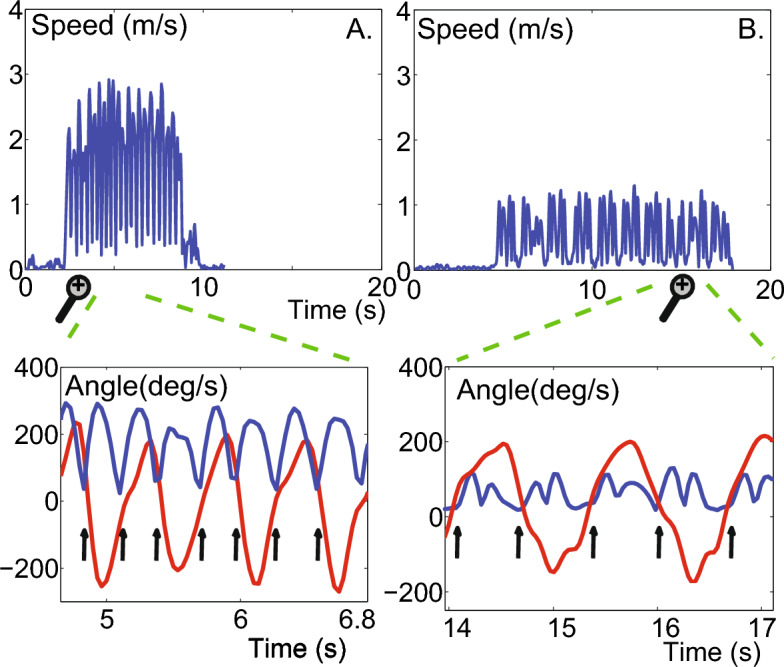
**IMAS camera and IMU data.** Examples of wrist speed profiles of an elbow flexion/extension task performed by two PD patients with different motor impairments. The speed profiles of the patient with lower motor impairment show clear speed minima (4.A), i.e., beginning and end of each movement, differently from the speed profiles of the patient with higher motor impairment (4.B) for which gyroscope recordings are needed to determine the start and stop of each movement. The bottom half of the Fig. shows an expanded view of the segmented movements (indicated by the black vertical arrows ) from the speed profile where the gyroscope data (red) is overlaid on the camera data (blue)

**Fig. 5:**
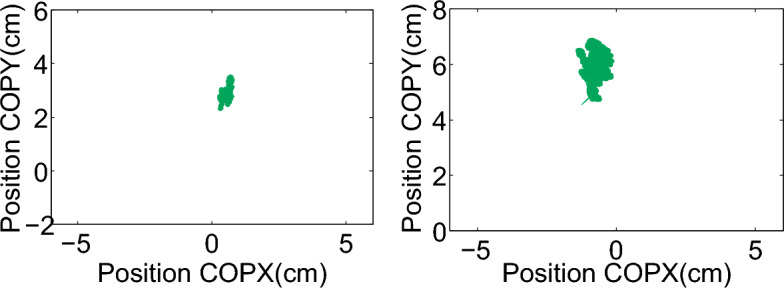
**IMAS force plate data.** Examples of two PD patients with different abilities to control body posture as measured by the force plate. CoP trajectories of a patient with UPDRS-III = 14 (path length = 23.57 cm) and of a patient with UPDRS-III = 46 (path length = 51.73 cm) are shown in the left and right panel, respectively

Figure 4 shows exemplary IMAS-derived data for two PD patients with UPDRS-III scores of 46 (Fig. 4B) and 14 (Fig. 4A), where a higher score indicates a higher impairment. The wrist speed profiles of the patient with score 46 are indicative of elbow flexion/extension movements that are slower and less smooth compared to the profiles of the patient with score 14 (mean speed = 0.51 m/s (0.06) vs. 1.6 m/s (0.21), standard deviation in parentheses; max speed = 1.10 m/s (0.12) vs. 2.5 m/s (0.33), and movement duration = 0.67 s (0.08) vs. 0.30 s (0.06); movement smoothness = 0.47 (0.07) vs. 0.64 (0.07)). Similarly, compared to the patient with UPDRS-III of 14, the patient with UPDRS-III of 46 moved more slowly during the hand opening/closing tests (average movement duration = 1.33 s vs. 0.47 s for the first test of this class, and 1.46 s vs. 0.48 s for the second); took longer for completing the complex, multi-joint motor tasks (43.13 s vs. 20.06 s respectively); and performed the hand-to-nose movements less easily (movement smoothness of 0.44 vs. 0.53; mean speed of 0.44 m/s vs. 0.89 m/s; max speed of 1.01 m/s vs. 1.68 m/s, on average). Additionally, the patient with the UPDRS-III of 46 showed a more prominent postural tremor, with 38.5% greater power than the patient with the UPDRS-III of 14 (2.75 vs. 1.98) as well as poorer postural control as shown by the CoP oscillations in Fig. 5 (for eyes open testing: path length = 51.73 cm vs. 23.57 cm; mean jerk = 0.11 m/s^3^ and max jerk = 0.40 m/s^3^ vs. mean jerk = 0.04 m/s^3^ and max jerk = 0.13 m/s^3^; for eyes closed testing: path length = 83.79 cm vs. 23.58 cm; mean jerk = 0.15 m/s^3^ and max jerk = 0.52 m/s^3^ vs. 0.03 m/s^3^ and 0.10 m/s^3^) and greater walking impairment (average total walking times of 15.75 s vs. 7.25 s, most affected leg average stride times of 1.24 s vs. 1.04 s, and stride counts of 13 vs. 6).

**Fig. 6:**
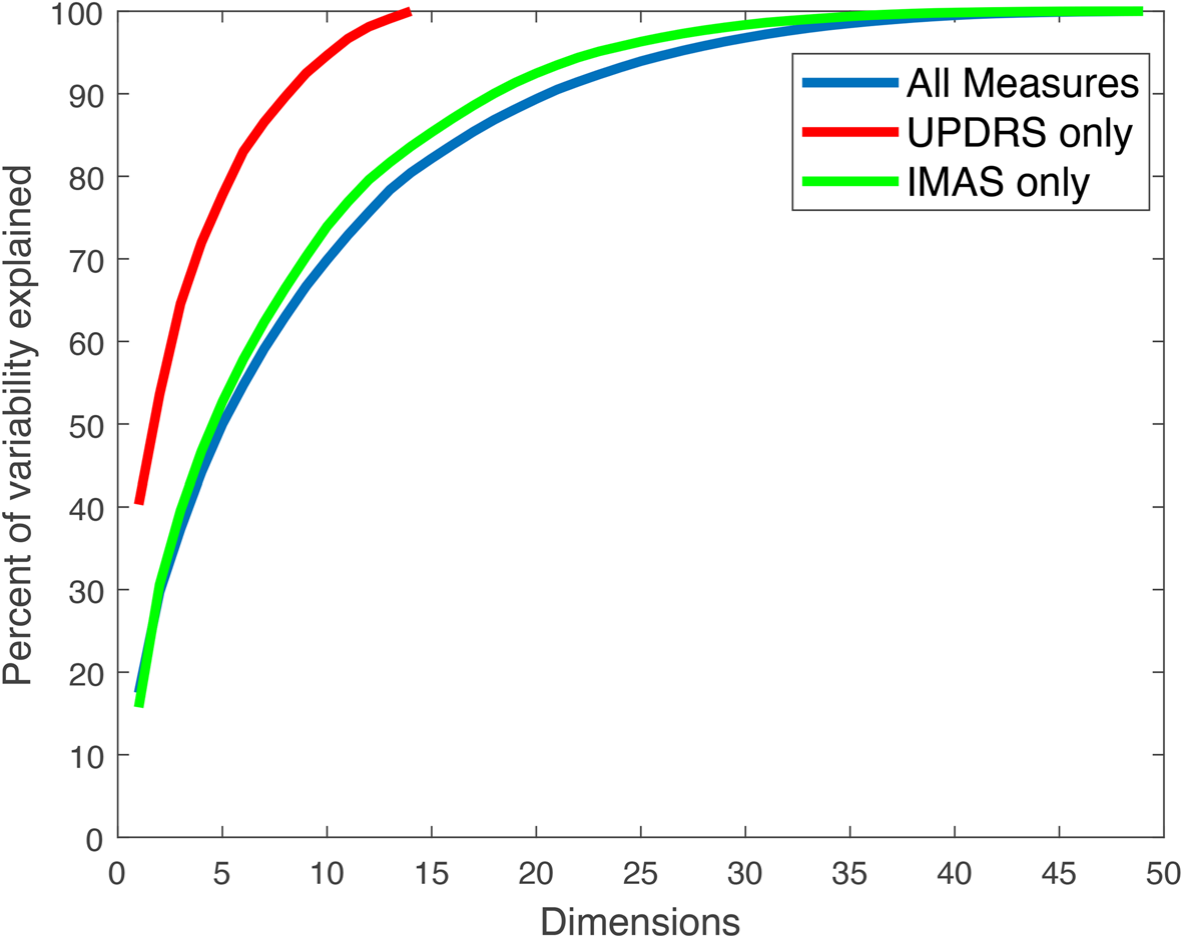
**PCA.** Percentage of variability as a function of the number of PCs retained for the UPDRS-III, IMAS, and combined data sets

Figure 6 shows the PCA results. Each line represents the percentage of the total variability among the given set of standardized signals as a function of the number of Principal Components (PCs) retained. The red line shows the PCA results for the set of UPDRS-III measures. The 1st PC captured around 40% of the variability in the data. An analysis of the PCs showed that the 1st PC had large positive contributions from all the UPDRS-III measures except for the two related to tremor (Q20, Q21), postural stability (Q30), and arise from chair (Q27). The posture and rise from chair were the largest positive contributors to the 2nd PC and the tremor questions to the 3rd PC. Additionally, the first 5 PCs captured ~ 80% of the variability. Although the UPDRS-III total score is the gold-standard for PD motor assessments, not surprisingly, these results provide evidence that there is additional variability in the UPDRS-III measures that is not explained by this score alone. The green line shows the PCA results for the IMAS metrics. The 1st PC alone explains ~ 20% of the total variability in these measures. An analysis of the PCs associated to the IMAS metrics showed that the 1st PC had the largest positive contributions from task time in continuous elbow flexion/extension, complex sequence of movements, and both hand opening/closing tasks and large negative contributions from the mean speed and peak speed during the hand to nose test and mean speed of discrete elbow flexion/extension movements. About 12 PCs were required to capture ~ 80% of the variability in these measures. This suggests that the effective number of independent dimensions associated with the IMAS measures is larger than that of the UPDRS-III measures. The blue line shows the PCA results when the UPDRS-III and IMAS measures are combined. The plot for the variance explained as a function of dimension for the combined dataset is consistently close to that of the IMAS alone, suggesting that adding data from the IMAS increases the number of independent measures beyond what is available from the UPDRS-III alone, but that adding the UPDRS-III data might not increase the number of independent measures from what is available from the IMAS data alone. Examining the 1st PC of the combined dataset, we found the weights associated with the UPDRS-III measures were close to the 1st PC of the UPDRS-III data alone, and that the weights associated with the IMAS measures were close to the 1st PC of the IMAS measures alone. This suggests that the combination of IMAS measures along which variability is maximal may be linearly predictive of the sum UPDRS-III measure.

The simplest prediction model, based on the elastic net regression, demonstrated an R^2^ of 0.54 and an MAE of 5.2 points of the total UPDRS-III score on the LOOCV evaluation. The key predictors identified from the elastic net based on the IMAS dataset included a mix of metrics from the 5 key movements (elbow flexion/extension (6 metrics), hand opening/closing (2 metrics), hand-touch-nose (6 metrics), tremor (5 metrics), modified Romberg (2 metrics), and walking (3 metrics)). IMAS signals with the largest weights in the elastic net model included peak speed of continuous elbow flexion/extension, movement duration and variability in movement mean speed of the hand-touch-nose task, path length of CoP oscillations during balance task with eyes closed, and path length of discrete elbow flexion/extension. For the assessments of N = 11 patients evaluated on day 2, the prediction results demonstrated an MAE of 4.32 points of the total UPDRS-III score and R^2^ = 0.75. Notably, the mean errors across our models are lower than past published values of inter-rater variability (23), which compared typical clinical staff to movement disorder experts and demonstrated mean errors in UPDRS-III evaluations of up to 5.4 points (see below for further examples).

Taken together, the PCA and modeling analysis suggest that the IMAS signals contain much of the information present in the UPDRS-III data and can predict the UPDRS-III score. The IMAS signals contain additional information not present in the UPDRS-III data which could be useful in identifying symptom patterns not typically captured in classic exams.

**Fig. 7:**
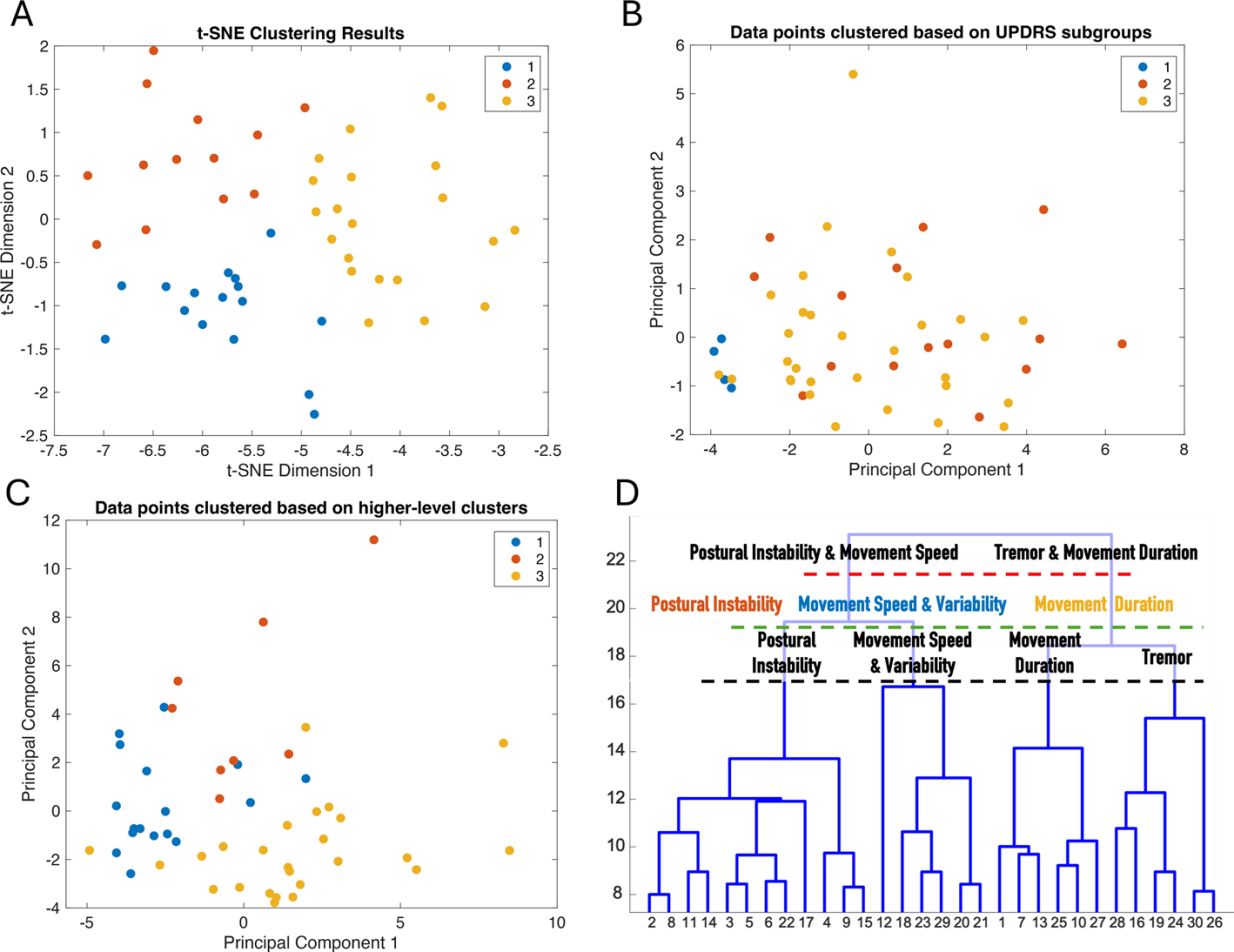
**Clustering. A** displays clusters identified by a K-means clustering algorithm from a dataset reduced via t-SNE (perplexity = 30, learning rate = 200, maximum number of iterations = 1000). The data points are plotted after dimensionality reduction by t-SNE and color-coded based on cluster membership, as per the K-means algorithm (3 desired clusters).** B** shows the classification of patients into 3 groups (1. tremor-dominant, 2. akinetic-rigid, and 3. mixed) based on their UPDRS-III scores, using the Eggers et al. method [149]. Each patient’s classification is depicted along the first two PCs derived from a PCA analysis of the UPDRS-III scores. Each group is distinctly color-coded according to the clinical classification.** C** illustrates the clusters obtained through higher-level clustering derived from the neuron weights of the SOM. The plot uses the first two PCs of the original input data, with data points color-coded according to their cluster identities, as per the hierarchical clustering algorithm. A comparative analysis of **A** and **C** with **B** shows that the IMAS metrics produce more clearly delineated and visually distinct clusters compared to the Eggers method based on UPDRS-III subscores. **D**: The dendrogram shows the results of hierarchical clustering using the Ward method on the SOM neuron weights. The y-axis represents the linkage distances, indicating the variance increase with each cluster merger. Clustering into 2, 3, and 4 groups highlight the structural relationships and significant separations within the data. For each group, the most important features are reported (see text). Note, the color coding for the 3 clusters corresponds to that in** C**

Figure 7, panels A-C depicts illustrative results of the clustering procedure. Various SOM configurations were systematically explored, including different grid sizes, numbers of epochs, and initial neighborhood sizes, and their impact on the quantization error was assessed. The optimal grid size of 6 × 6 was identified based on the lowest error, and subsequently trained 10 times to evaluate the model’s stability. The most effective SOM configuration was selected based on the lowest quantization error. The Ward method was then employed for hierarchical clustering. These clustering results are in line with the PCA results. Clusters based on IMAS measures (a dataset richer than the UPDRS-III data set, as indicated by PCA) are more separable/distinct than those based on UPDRS-III. The higher discriminative power of the IMAS-based clusters indicates that the IMAS-based features capture meaningful differences across the dataset, which might facilitate personalization of treatments as patients within each cluster are more homogeneous in terms of how they might respond to a specific treatment.

A random forest algorithm was used to explore the IMAS features that most contributed to each cluster. The algorithm was trained across 10 iterations, each with a different random seed to ensure variability, using out-of-bag predictor importance to assess the stability of feature importance scores. The top ten most important features were selected based on having the highest average importance scores. Figure 7, panel D displays these features in the context of the clusters selected by the Ward algorithm, specifically for 2, 3, and 4 clusters. The dendrogram visualizes how these clusters are related and their separation based on the IMAS features. Notably, the clustering effectively distinguishes between patient groups characterized by postural instability, tremor, and bradykinesia metrics. These clusters align with recognized symptom patterns of PD, while also potentially providing insights for treatment planning. Clinical subgroup classifications such as those explored by Eggers et al. [149] often distinguish between tremor-dominant and akinetic-rigid patients, relegating patients without tremor or bradykinesia/rigidity as dominant traits, to a mixed group. However, the IMAS-based clusters highlight the importance of postural instability subgroups, such as in [150–152], which might provide a basis for tailoring treatments. While postural instability was believed to herald a late-stage PD, it is now known to be a dominant phenotypical trait in early PD [151]. Its early diagnosis may be elusive in clinical exams and hinders PD patients’ wellbeing. Although research initiatives focused on CoP or center of gravity measures alone have not been adopted in clinical practice, this finding might find a more direct application because postural and gait instability cause PD patients to fall, which is a main driver for morbidity and has become the main culprit raising health care expenditures amongst PD motor symptomatology [153]. Additionally, of note is the evolving clinical definition of bradykinesia [154] and the potential for IMAS clustering to identify testing metrics for patient subgroup classification. Finally, the prediction model built on different clusters showed improved prediction accuracy compared to the simple prediction model described above. For example, a prediction model based on the 3 clusters achieved an MAE of 4.34 points and an R^2^ of 0.7 and a model based on the 4 clusters achieved an MAE of 4.17 and an R^2^ of 0.65 via LOOCV.

### Integration in the PD care pathway

While this section has presented results from the IMAS in a cohort of 50 PD patients, IMAS is specifically designed to systematically collect voluminous datasets of motor behavioral data during patient assessments, as depicted in Fig. 1–3. Assessments are a crucial part of the PD care continuum, and IMAS is engineered to integrate at various stages within this continuum. During periodic assessments, IMAS can aid in patient evaluation and can also be trained to output traditional evaluation scores. While we have reported results for UPDRS-III, the algorithms can be trained to generate other clinical scores, such as MDS-UPDRS-III. Other notable features of IMAS include the capability of its learning core to be trained on different evaluators (such as a specific senior movement disorder expert at a clinical practice, who for instance could be used to train or calibrate other staff), and the potential for its integration with telemedicine, which enables assessments of patients in remote locations or those lacking access to specialists.

**Fig. 8:**
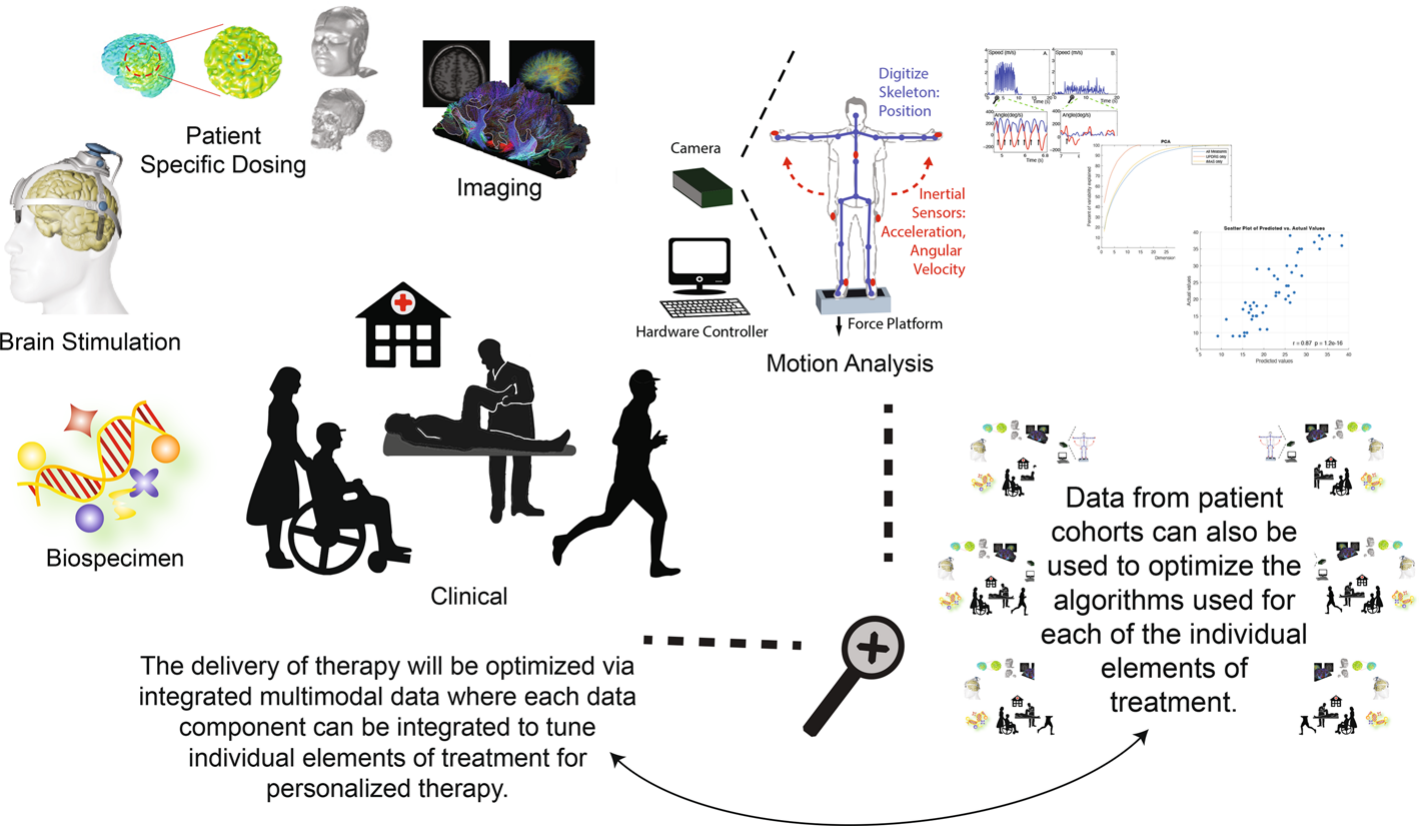
**IMAS future expansion and data integration.** Planned IMAS implementation and integration with additional data types (e.g., biospecimen, imaging, etc.). This figure is adapted from our Fig. 5 in [6]

Treatment selection or adjustment is another stage of the care continuum where IMAS can have a significant impact. Traditional methods often rely on trial and error and clinical subgroup classifications based on the UPDRS scores or clinical history, such as those of [149, 155]. However, these types of classifications share the same limitations as UPDRS, providing only coarse clustering. IMAS-based clusters can be further enhanced by integrating additional data types, such as for example features extracted from neuroimaging, biospecimen, and/or autonomic data (e.g., blood pressure, pupillary response). The applications are numerous; they can aid in developing personalized treatment plans tailored to specific patient symptoms and likely disease progression. As new treatments (e.g., neuromodulation techniques) and more traditional treatments (e.g., PT) are developed or further explored, they can facilitate understanding of how these interventions impact the motor system over time and help build methods for optimizing such interventions- See Fig. 8. Finally, with the evolving understanding of PD, and the continued identification of clinical subtypes and disease processes (e.g., [152, 154, 156–159]), IMAS might not just help with treatment selection, but could be used to enhance our fundamental understanding of the disease and/or be used as a tool to aid initial diagnosis.

## Conclusion

Big Data is changing clinical research and care. A central challenge is integrating the massive amounts of data that are increasingly collected through modern technologies, such as wearable and portable systems, watches, and smartphones with traditional health care. This data must be combined with traditional clinical data that clinicians are accustomed to collecting and can readily interpret. Finding effective ways to integrate this data is crucial for developing technologies that can make a significant impact in the clinical world and ultimately improve patients' lives.

This paper presented a potential approach to address this challenge. We focused on PD, a global health concern that urgently requires the development of new methodologies, possibly involving advanced technologies. Among these, Big Data and AI hold enormous, yet largely untapped, transformative potential. We showed how sensor-based data, increasingly used in patient monitoring and collected herein with the IMAS, can be integrated with traditional motor assessment data. Using a hybridization approach [160], we combined elements from existing solutions to develop a system that outperforms each individual component. Specifically, we showed that IMAS measures: 1) exhibited higher effective dimensionality compared to the measurements that comprise traditional UPDRS-III scores and were able to explain most of the variability of UPDRS-III, suggesting that the IMAS measures contain a richer representation in their underlying data structure, encompassing a broader spectrum of information and potentially more nuanced insights, and have the potential for predicting UPDRS-III; 2) enabled predictions of UPDRS-III scores; and 3) enabled the identification of distinct patient clusters. These clusters not only improved UPDRS-III predictions but were also clinically interpretable and could potentially provide a basis for tailoring treatments to meet the specific needs of different patient groups.

The use of multimodal sensors in IMAS overcomes the limitations of individual sensor modalities, enabling a comprehensive assessment of motor function. IMAS stands out from existing systems in the literature by integrating multiple data sources into a unified system that generates clinically interpretable outputs and is applicable in real-world settings. While many systems focus solely on data collection or analysis, IMAS bridges the gap between research innovation and clinical adoption, making it viable across the PD care continuum. We demonstrated several potential uses for IMAS across the care continuum and by various members of the PD care team. For example, primary care providers could use the IMAS to aid in early diagnosis with augmented UPDRS-III assessments; physical therapists could use the IMAS to complete more refined motor assessments during rehabilitation exercises; movement disorder experts could use IMAS clustering methods to study fundamental disease traits not clearly resolved with traditional methods; and technologists could use the IMAS to tune device parameters, such as non-invasive brain stimulation doses, for optimal treatment outcomes. Moreover, IMAS is designed so that the physician workload can be minimized by allowing other members of the care team (e.g., physician assistants, technicians) to facilitate patient evaluations. Furthermore, although not the focus herein, IMAS has also been designed with the understanding that its adoption depends on its ability to garner and facilitate reimbursement, EHR integration, and data privacy [161] in a manner that can improve the cost-effectiveness of patient management [6].

While our approach focuses on PD and was based on IMAS, validated herein for PD motor assessments, it can be generalized to other contexts where integration issues arise (of note, we have explored the IMAS in stroke and chronic pain conditions, such as diabetic neuropathic pain, carpal tunnel syndrome pain, and lower back pain). Furthermore, we discussed the importance of defining a path for integrating advanced technologies into the clinical care pathway and exemplified a solution by demonstrating how IMAS can be effectively incorporated into the PD care pathway.

While Big Data, AI, and advanced technological infrastructures are rapidly transforming clinical research, the challenge of integrating innovations into routine clinical practice remains significant. IMAS not only advances the state-of-the-art but also offers a practical model for how such technologies can be adopted in clinical settings. Future efforts should prioritize overcoming the technical and practical barriers to ensure that advanced methodologies like IMAS are seamlessly incorporated into established clinical workflows.

## Data Availability

The clinical trial data is planned for upload to clinicaltrials.gov following the completion of ongoing follow-on trials. The full dataset and software generated and analyzed during the current study are not publicly available as proprietary and restricted by intellectual property rights but data are available from the corresponding author on reasonable request, subject to necessary approvals.

## Abbreviations

PD: Parkinson’s Disease
AI: Artificial intelligence
IMAS: Integrated Motion Analysis Suite
UPDRS: Unified Parkinson’s Disease Rating Scale
PCA: Principal component analysis
U.S.: United States
PT: Physical therapy
PC: Principal Component
MDS: Movement Disorder Society
DBS: Deep brain stimulation
RGB-D: Red Green Blue-Depth
CoP: Center of pressure
EMG: Electromyography
PIGD: Postural instability and gait difficulties
MAE: Mean absolute error
CNN: Convolutional neural network
LOOCV: Leave one out cross validation
RMSE: Root mean square error
ADL: Activity of daily leaving
EHR: Electronic health record
SOM: Self organizing map
SRH: Spaulding Rehabilitation Hospital
IMU: Inertial measurement unit
DF: Degree of freedom
t-SNE: T-distributed stochastic neighbor embedding
